# Supplementary material for: Two Ascophyllum nodosum Fucoidans with Different Molecular Weights Inhibit Inflammation via Blocking of TLR/NF-κB Signaling Pathway Discriminately
Source: Foods. 2022 Aug 8;11(15):2381. doi: 10.3390/foods11152381 (PMC9368091; doi:10.3390/foods11152381)
Supplement: Supplementary file 1 [file foods-11-02381-s001.zip › foods-1811087-supplementary.pdf]

Two Ascophyllum nodosum Fucoidans with Different Molecular Weights Inhibit Inflammation via Blocking of TLR/NF-κB Signaling Pathway Discriminately

Lilong Wang[1], Linlin Wang[1], Chunhong Yan[1], Chunqing Ai[1], Chengrong Wen[1], Xiaoming Guo[2], Shuang Song[1,*]

[1] National Engineering Research Center of Seafood, School of Food Science and Technology, Collaborative Innovation Center of Seafood Deep Processing, School of Food Science and Technology, Dalian Polytechnic University, Dalian 116034, P. R. China

[2] Shenzhen Key Laboratory of Food Nutrition and Health, Institute for Advanced Study, Shenzhen University, Shenzhen, 518060, P. R. China

* Corresponding author at School of Food Science and Technology, Dalian Polytechnic University, No.1 Qinggongyuan, Ganjingzi District, Dalian 116034, P. R. China. Tel: +86-411-86323262, Fax: +86-411-86323262. E-mail address: songs1008@163.com.

Table S1. Primer sequences used for quantitative RT-PCR reactions.

Gene symbol	Forward primer sequence	Reverse primer sequence
TLR2	CAGCTGGAGAACTCTGACCC	CAAAGAGCCTGAAGTGGGAG
TLR4	CAACATCATCCAGGAAGGC	GAAGGCGATACAATTCCACC
iNOS	ATGTCCGAAGCAAACATCAC	TAATGTCCAGGAAGTAGGTG
COCX2	CAGCAAATCCTTGCTGTTCC	TGGGCAAAGAATGCAAACATC
TNF-α	GATCGGTCCCCAAAGGGATG	GGCTACAGGCTTGTCACTCG
IL-1β	TTCATCTTTGAAGAAGAGCCCAT	TCGGAGCCTGTAGTGCAGTT
IL-6	TGGAAATGAGAAAAGAGTTGTGC	CCAGTTTGGTAGCATCCATCA
IL-10	TACTCGGCAAACCTAGTGCG	GTGTCCCAACATTCATAATTGTCAGT
β-actin	TCAGCAAGCAGGAGTACGATG	AACGCAGCTCAGTAACAGTCC
